# The oral nucleoside drug VV116 is a promising candidate for treating Nipah virus infection

**DOI:** 10.1080/22221751.2025.2587983

**Published:** 2025-11-19

**Authors:** Yumin Zhang, Yanfeng Yao, Shufen Song, Ge Gao, Yun Peng, Hang Liu, Miaoyu Chen, Wei Zheng, Guanghui Tian, Yuanchao Xie, Jingshan Shen, Gengfu Xiao, Tianwen Hu, Chao Shan, Leike Zhang

**Affiliations:** a State Key Laboratory of Virology and Biosafety, Wuhan Institute of Virology, Center for Biosafety Mega-Science, Chinese Academy of Sciences, Wuhan, People’s Republic of China; b University of Chinese Academy of Sciences, Beijing, People’s Republic of China; c Vigonvita Shanghai Co., Ltd, Shanghai, People’s Republic of China; d State Key Laboratory of Drug Research, Shanghai Institute of Materia Medica, Chinese Academy of Sciences, Shanghai, People’s Republic of China; e Lingang Laboratory, Shanghai, People’s Republic of China

**Keywords:** VV116, Nipah virus, oral nucleoside drug, antiviral effect, hamster model

## Main text

Nipah virus (NiV) is an emerging pathogen belonging to the Henipavirus genus within the family Paramyxoviridae. This single-stranded, negative-sense RNA virus can be transmitted directly from fruit bats (genus *Pteropus*) to humans or through intermediate hosts such as pigs or horses. NiV causes acute respiratory illness and fatal encephalitis with a mortality rate ranging from 40% to 70% [1,2]. The virus was first identified during an outbreak in Kampung Sungai Nipah, Malaysia between 1998 and 1999 [1], and was later reported in Bangladesh and India in 2001 [3,4]. Based on geographic distribution, two divergent NiV strains have been designated: NiV-Malaysia (NiV-M) and NiV-Bangladesh (NiV-B). Initially, human infections in Malaysia primarily presented with encephalitic symptoms, while pigs exhibited respiratory issues. However, subsequent outbreaks in Bangladesh, India, and the Philippines revealed respiratory symptoms in humans, indicating a significant potential for human-to-human transmission. Due to its broad host range (including bats and livestock), high case-fatality rate, and the absence of approved drugs or vaccines, the World Health Organization (WHO) classified NiV as a highest-priority regional threat in 2018. Furthermore, regions in Southeast Asia are particularly vulnerable due to high population density, the presence of *Pteropus* fruit bats, and frequent interactions between animals and humans, all of which increase the risk of NiV transmission. Although several small-molecule compounds or monoclonal antibodies (e.g. ERDRP-0519, DS90-m102.4) have demonstrated effective inhibition of NiV in experimental settings [5,6], no drugs have yet been licensed for clinical use. Therefore, there is an urgent need to develop therapeutic interventions against NiV for both humans and animals.

We previously reported an oral nucleoside prodrug, VV116, which demonstrated antiviral potency against SARS-CoV-2 variants and other human coronaviruses both *in vitro* and *in vivo* [7,8]. Due to its positive outcomes in clinical trials [9,10], VV116 has been approved for the treatment of COVID-19 in China and Uzbekistan. Additionally, VV116 has been shown to potently inhibit respiratory syncytial virus (RSV) in mice [11], however, its antiviral activity to NiV remains unknown.

**Figure 1.**
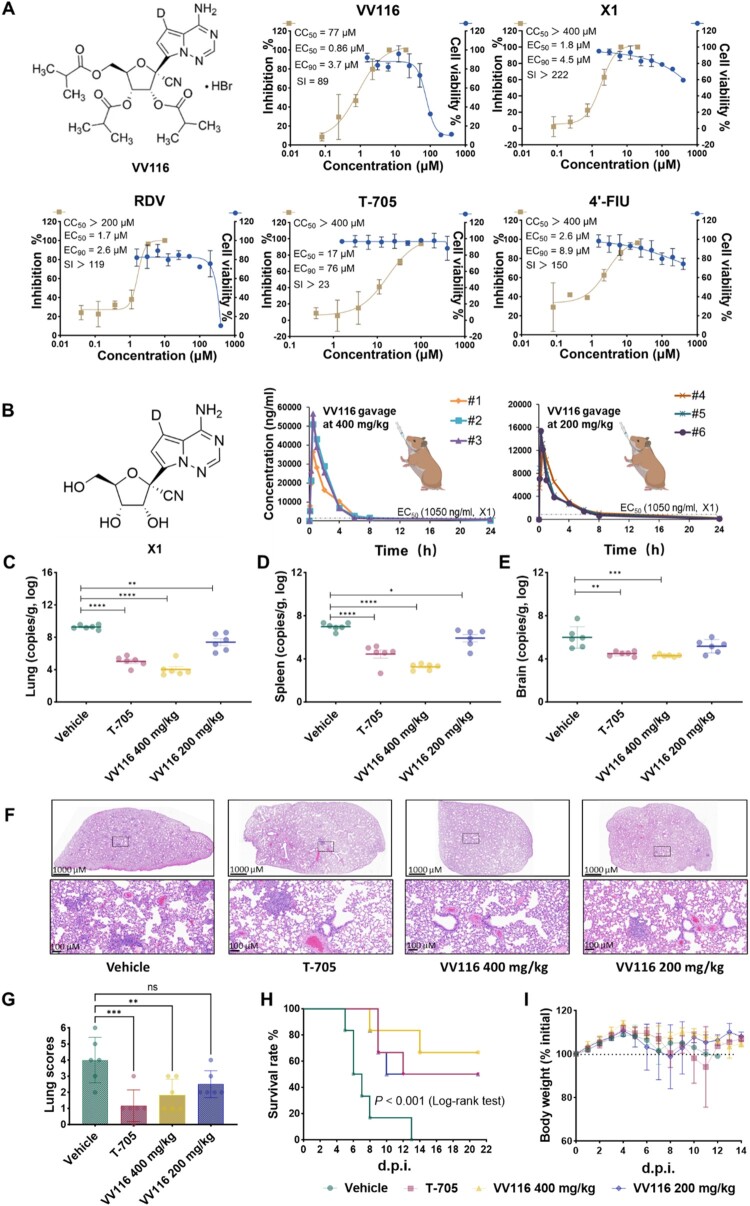
The oral nucleoside drug VV116 effectively inhibits NiV-M both *in vitro* and *in vivo*. **A** Chemical structure of VV116, along with the antiviral activity and cellular toxicity of VV116, X1, RDV, T-705, and 4’-FlU in Vero E6 cells. Error bars represent the standard deviation (SD) of three independent experiments. **B** Concentration of X1 in blood following oral administration of VV116 at single doses of 400 mg/kg or 200 mg/kg in hamsters (*N* = 3 per each dose group). **C-E** Viral RNA copies detected in lung (**C**), spleen (**D**), and brain tissues (**E**) of vehicle-, T-705-, and VV116- (400 and 200 mg/kg) treated hamsters at 4 d.p.i. (*N* = 6). T-705 was treated at 600 mg/kg on the day of viral challenge and subsequently at 300 mg/kg once daily. **F** Histopathology of hamster lungs from vehicle, T-705, VV116-400 mg/kg, and VV116-200 mg/kg treatment groups. Imagines in the lower panel are enlarged views of those in the upper panel. **G** Histopathology scoring of lungs: scores 1–2 indicate mild, 3–4 moderate, and 5–6 severe pathology. **H-I** The survival rates (**H**) and body weight changes (**I**) of hamsters in different treatments (*N* = 6). Error bars in *in vivo* experiments represent the standard error of the mean (SEM). Statistical significance between groups was determined by the log-rank test (survival rates) and one-way ANOVA (viral RNA quantification). *p < 0.05, **p < 0.005, ***p < 0.0005, ****p < 0.0001.

To investigate whether VV116 is a potential drug candidate for treating NiV infection, we first assessed the anti-NiV activity of VV116 *in vitro* (Figure 1A). In Vero E6 cells, VV116 and remdesivir (RDV) showed comparable EC_50_ values of 0.86 µM (SI = 89, Selectivity Index) and 1.67 µM (SI > 119) against NiV-M, respectively. Compound X1, the parent nucleoside analog and metabolic form of VV116, has the EC_50_ value at 1.8 µM. Given the reported anti-NiV activity of favipiravir (T-705) and 4’-fluorouridine (4’-FlU) [12,13], we conducted a head-to-head comparison of their activities in our cell-based assay. The results exhibited that VV116, RDV, and X1 were more potent against NiV-M than T-705 and 4’-FlU, which had EC_50_ values of 17 µM (SI > 23) and 2.6 µM (SI > 150), respectively. The C_max_ of X1 following a single 800 mg oral dose of VV116, as determined in a Phase I clinical trial, was approximately 4.8 µM (2,796 ± 225 ng/ml) [14], a concentration exceeding the EC_90_ value of X1 for inhibiting NiV-M in Vero E6 cells. Additionally, VV116 and X1 effectively inhibited the NiV-B strain with EC_50_ values of 0.97 and 1.17 µM, respectively (Figure S1). To evaluate the preclinical therapeutic potential of VV116 against NiV infection *in vivo*, we conducted a pharmacokinetic (PK) study of VV116 in golden Syrian hamsters. VV116 exhibited a linear PK profile at doses of 200 and 400 mg/kg (Figure 1B). Following oral administration, the blood concentration of the parent nucleoside X1 reached C_max_ within 1 h, with half-lives (T_1/2_) ranging from 3.9 to 6.0 h. VV116 showed high plasma exposure with C_max_ of 48,067 ± 10,484 ng/ml and AUC_0-t_ of 124,174 ± 15,830 h·ng/ml at a single oral dose of 400 mg/kg (**Table S1**). Furthermore, metabolite X1 was widely distributed in lung and spleen after oral administration of VV116 at 400 mg/kg, with concentrations of 24,600 and 27,967 ng/ml, respectively (Figure S4). These drug concentrations in the lung and spleen are comparable to plasma levels. X1 was also detected in brain, although at concentrations approximately 20-fold lower than in the other target organs. Given that viral loads in the brain were about 1–3 logs lower than those in the lung and spleen (Figure 1C-E), it explains that the penetration of drug into the brain was sufficient to suppress viral replication. The favourable PK profile and potent *in vitro* antiviral activity of VV116 support its potential efficacy against NiV infection *in vivo*.

Therefore, we further evaluated the *in vivo* antiviral activity of VV116 against NiV in a hamster model. The flow diagram of the *in vivo* antiviral experiments is shown in Figure S2. Female golden Syrian hamsters were intraperitoneally infected with NiV-M at a lethal dose (8.55 × 10^3^ TCID_50_, 1000 LD_50_). VV116 was orally administered once daily at doses of 200 or 400 mg/kg for 14 days. The buffer used as the drug solvent (vehicle) and T-705 (600 mg/kg on the day of viral challenging, followed by 300 mg/kg once daily for 13 days) treatments served as controls. Initial drug or vehicle treatments were administered at 1 h post-infection (h.p.i.). On day 4, animals were euthanized, and target tissues of viral infection were collected for viral load quantification and histopathological analysis. The results showed that VV116 exhibited a dose-dependent efficacy in reducing viral RNA copies in lungs, spleens, and brains (Figure 1C-E). The 400 mg/kg VV116 treatment group decreased viral RNA copies by approximately 4, 2, and 1.5 logs in the lungs, brains, and spleens, respectively, compared to the vehicle group. The lower dose (200 mg/kg) also significantly reduced viral loads, except in the brain. As a control, T-705 treatment (600 mg/kg + 300 mg/kg) effectively inhibited NiV-M replication in hamsters, consistent with previous reports. Immunofluorescence staining for NiV-M F protein confirmed viral load reduction in tissues from the VV116 and T-705 treatment groups compared to the vehicle group (Figure S3). Histopathological examination of NiV-M–infected lung tissues revealed thickened alveolar septa, infiltration of inflammatory cells, and significant inflammatory reactions in blood vessels. In contrast, lungs from the 400 mg/kg VV116 treatment group showed no significant histopathological abnormalities, while lungs from the T-705 and 200 mg/kg VV116 groups exhibited mild lesions (Figure 1F-G).

To evaluate the protective effect of VV116 on hamsters infected with NiV, the same oral drug or vehicle treatments described above were administered following intraperitoneal infection with 1000 LD_50_ of NiV-M. Drugs or vehicle were orally administered once daily for 14 days. The animals were monitored daily for food and water intake, disease signs, changes in body weight, and survival rates. The results showed that vehicle-treated hamsters had a 30% survival rate at 7 d post-infection (d.p.i.), then all deceased by 13 d.p.i.. In contrast, all drug treated groups exhibited a 100% survival rate at 7 d.p.i., although the survival rates were 50% for T-705, 50% for VV116-200 mg/kg, and 66.7% for VV116-400 mg/kg by the endpoint (21 d.p.i.) (Figure 1H). All NiV-infected hamsters, regardless of treatment, began losing body weight at 4 d.p.i., but only those treated with VV116 at 400 mg/kg maintained body weights above their initial levels through the final day (Figure 1I). The disease signs in the vehicle group appeared at 4 d.p.i., including reduced activity, decreased appetite, piloerection, and arched backs, and these signs were delayed in the drug-treated groups. Our *in vivo* experiments demonstrate that oral administration of VV116 exhibits potent anti-NiV activity.

## Discussion

Small-molecule drugs are generally more accessible than biologics due to their enhanced stability, lower cost, and the feasibility of oral formulation. Currently, only a limited number of nucleotide analogs have demonstrated efficacy against NiV both *in vitro* and *in vivo* studies. RDV has exhibited broad-spectrum antiviral activity against RNA viruses, including coronavirus and paramyxovirus [15,16], and has been shown to protect African green monkeys from severe respiratory disease induced by NiV [17]. Additionally, 4’-FlU has demonstrated antiviral efficacy against multiple hemorrhagic fever viruses as well as NiV [13]. Our results showed that VV116, X1, RDV, and 4’-FlU exhibited comparable anti-NiV-M activity *in vitro*. T-705, used as a control compound in the *in vivo* antiviral experiments in this study, has been reported to inhibit NiV with an effective concentration (EC_50_) range of 14–44 µM in Vero cells. Twice-daily oral administration of T-705 at 300 mg/kg (600 mg/kg on the challenge day) over a 14-day period provided protection to hamsters infected with a lethal dose of NiV [12]. Although the detailed mechanism by which VV116 inhibits NiV requires further investigation, we speculate that its active metabolite, X1, blocks viral replication by targeting the viral RNA-dependent RNA polymerase (RdRP), similar to the mechanism observed in anti-coronavirus activity [18]. In this study, we found that oral administration of T-705 at 400 mg/kg and VV116 at 200 mg/kg once daily prevented mortality in 50% of hamsters. Pharmacokinetic (PK) studies of VV116 at 200 mg/kg in hamsters indicated that plasma concentrations of X1 remained above the EC50 value for at least 8 h. As expected, a higher dose of VV116 (400 mg/kg) resulted in improved antiviral activity and survival protection. These findings suggest that the nucleoside drug VV116 is a very promising oral candidate against NiV infection. Based on a previous phase I clinical study [14], a dosage regimen of 800 mg twice a day and above can continuously maintain effective antiviral concentration and is recommended for subsequent clinical studies in patients with NiV infection. Due to the safety and favourable PK have been robustly evidenced in humans, VV116 should be considered as a therapeutic option for future NiV outbreaks and as a potential backup for individuals at high risk of exposure, such as healthcare staff and laboratory workers.

## Supplementary Material

20251017_NiV VV116 SI EMI_clean.docx
