# Supplementary material for: The oral nucleoside drug VV116 is a promising candidate for treating Nipah virus infection
Source: Emerg Microbes Infect. 2025 Nov 19;14(1):2587983. doi: 10.1080/22221751.2025.2587983 (PMC12632212; doi:10.1080/22221751.2025.2587983)
Supplement: 20251017_NiV VV116 SI EMI_clean.docx [file TEMI_A_2587983_SM4522.docx]

**Materials and Methods**

**Cells and Viruses**

Vero E6 (ATCC CRL-1586) was maintained in Dulbecco’s modified Eagle’s medium (DMEM) with 10% fetal bovine serum (FBS) and 1% penicillin-streptomycin antibiotics. Cells were cultured at 37 ℃ in a 5% CO_2_ atmosphere. The NiV-M (GenBank ID: AF212302) and NiV-B (GenBank ID: AY988601.1) strains employed in this study was obtained from the National Virus Resource Center, Chinese Academy of Sciences (CAS). NiV propagation was performed in Vero E6 cells in DMEM with 2% FBS. All experiments with authentic NiV-M virus were performed in the BSL-4 facility at the National Biosafety Laboratory, CAS.

**Antiviral activity and cytotoxicity measurements in cells**

To determine the anti-NiV-M and -NiV-B activity of compounds *in vitro*, Vero E6 cells were pre-seeded to 48-well plates overnight to 70-80% confluence, then the culture medium was removed and replaced with a medium containing a gradient concentration of compounds for 1 h incubation. Subsequently, cells were then inoculated with the NiV-M or NiV-B strain at a multiplicity of infection (MOI) of 0.01. At 96 h after infection, the supernatant was collected for viral RNA copy number determination using real-time fluorescence quantitative PCR (qRT-PCR, One-Step qRT-PCR SYBR Green Kit, Vazyme). The primers of qRT-PCR of NiV were: NiV-F2: 5’-GTGAGCAATCTGGTAGGAT-3’ and NiV-R2: 5’-TGACACTGCACAAAGCACA-3’. The viral RNA copy was calculated by standard plasmid concentration. The inhibition rate of compounds was calculated based on the viral copy number, and the 50% effective concentration (EC_50_) was calculated with GraphPad Prism software 9.0. These experiments were independently performed three times.

Cell viability was performed in 96-well plate with triplicate for each concentration using Cell Counting Kit-8 (CCK-8) assay. All drugs were diluted 2 times with 9 gradients starting at 500 micromoles in maintenance medium (DMEM containing 2% FBS). After 48 h incubation, the supernatant was removed, and 10 μL WST-8 (2-(2-methoxy-4-(phenyl)-3-(4-(phenyl) to 5 (2, 4-sulpho benzene) -2 h-tetrazolium monosodium salt) in maintenance medium was added in medium. Plates were measured at 450 nm wavelength using spectrophotometer (BioTek) after 2 h incubation, and cell viability was calculated. Three independent experiments were performed and the 50% cell cytotoxicity (CC_50_) was calculated with GraphPad Prism software 9.0.

**Pharmacokinetic study of VV116 in hamsters**

Nine golden Syrian hamsters (*N*= 3 for each group, female) were randomly divided into three groups, and fasted for 12 h before dosing. The two groups received oral dose of VV116 at 400 mg/kg and 200 mg/kg, respectively. The vehicle for oral administration of the test compounds was 5% DMSO+ 5% Solutol HS-15+5% PEG400+ 85% Saline. Blood sample (70 μL) was collected from the orbit of the hamsters in each group at 0.083, 0.25, 0.5, 1.0, 2.0, 4.0, 6.0, 8.0 and 24 h post-dosing. The sample was taken into EDTA-K2 tubes, and centrifuged at 11,000 rpm for 5 min. The plasma was separated and frozen in a refrigerator at −70 °C for testing. The operation was conducted under an ice water bath. The concentration of analyte X1 in plasma was determined by LC-MS/MS.

***In vivo* efficacy of VV116 against NiV-M**

Age of 5-6 weeks golden Syrian hamsters were purchased from Charles River Laboratory (Beijing). The animal experiments conformed to the use and care of laboratory animals and were approved by the ethics committee of Wuhan Institute of Virology, CAS (WIVA42202312). Animals were randomly divided into four groups (*N* = 6 each group), the vehicle group, the group receiving T-705, the group receiving VV116 400 mg/kg, the group receiving VV116 200 mg/kg. Hamsters were anesthetized by isoflurane inhalation and then intraperitoneally infected with 8.55×10^3^ TCID_50_ (1000 LD_50_) of NiV-M. One hour after viral infection, hamsters were orally treated with vehicle or drugs (day 0). Hamsters were treated once daily in the following days. On day 4, hamsters were sacrificed, and the lung, spleen, and brain tissues were collected for viral copies detection and histopathology study. Viral RNA from the tissues was extracted with the RNeasy Mini Kit (Qiagen), then the absolute viral RNA copy was determined quantitatively by One-Step qRT-PCR SYBR Green Kit (Vazyme Q221) with the primers of NiV-F2/R2 described above. For histological examination, tissues were collected after euthanasia and placed in 4% paraformaldehyde for fixation. Fixed tissue samples were used for hematoxylin-eosin (H&E) and immunofluorescence staining for the detection of the NiV-M antigen (NiV-M Fusion Protein Rabbit pAb, gifted by Professor Shan). The image information was collected using a Pannoramic MIDI system (3DHISTECH, Budapest) and FV1200 confocal microscopy (Olympus). The F protein signals were quantified as signal counts using Image J software (version 1.8, NIH).

To investigate whether VV116 treatments improve the survival rate of NiV-M infected hamsters, golden Syrian hamsters were divided into four groups (*N* = 6 each group) and treated with vehicle, T-705, and VV116 as same as the administration mentioned above. The hamsters were treated and observed daily for changes in body weight until day 14, and the survival rates were observed until day 21.

**Statistics**

All cell and animal data analyses and graphs were performed using GraphPad Prism software version 9 (GraphPad Software Inc., San Diego, CA). One-way ANOVA (analysis of variance) with Dunnett’s post-hoc test was used to determine statistical significance.

**Figure S1** Dose-dependent curves showing the activity of VV116, X1, RDV, T-705, and 4’-FlU against the NiV-B strain in Vero E6 cells. Error bars represent the mean ± standard deviation from three independent experiments. The EC_50_ values were calculated using nonlinear regression with GraphPad Prism software 9.0.

**
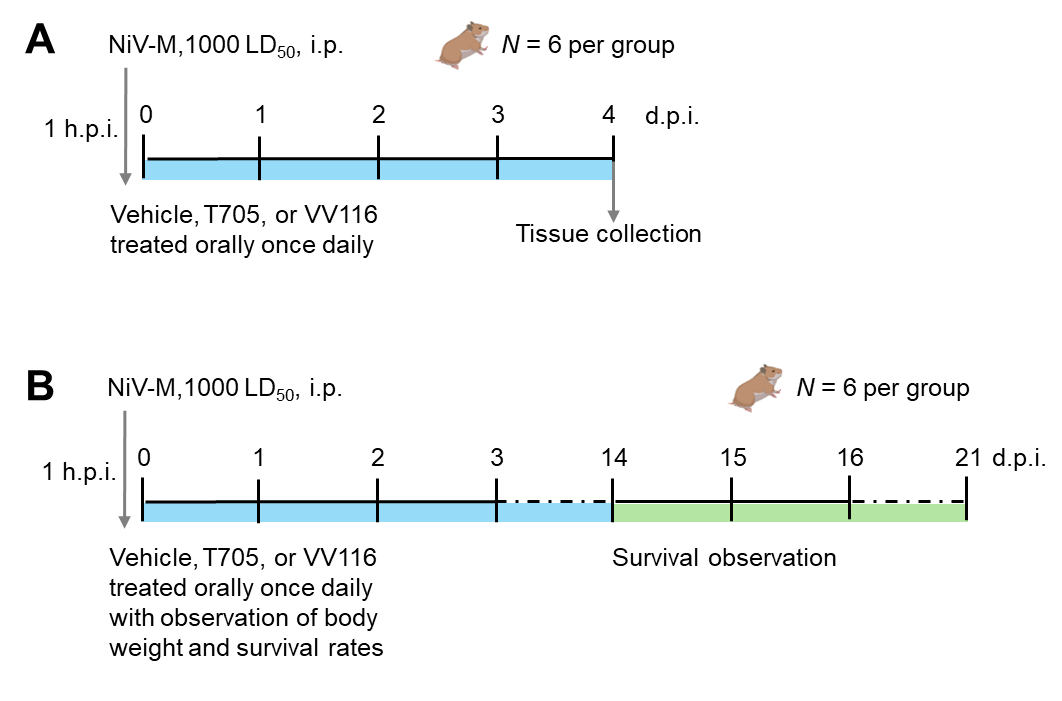
Figure S2** Flow diagram of *in vivo* antiviral experiments in hamsters. Hamsters were randomly divided into four groups (*N* = 6 each group), the vehicle group, the group receiving T-705, the group receiving VV116 at 400 mg/kg, and the group receiving VV116 at 200 mg/kg. Hamsters were intraperitoneally infected with 1000 LD_50_ of NiV-M. One hour after viral infection, hamsters were orally treated with vehicle or drugs (day 0). **A** Hamsters were sacrificed on day 4 and the lung, spleen, and brain tissues were collected for viral copies detection and histopathological analysis. **B** The hamsters were treated and monitored daily for changes in body weight until day 14, and survival was continuously observed until day 21.

**
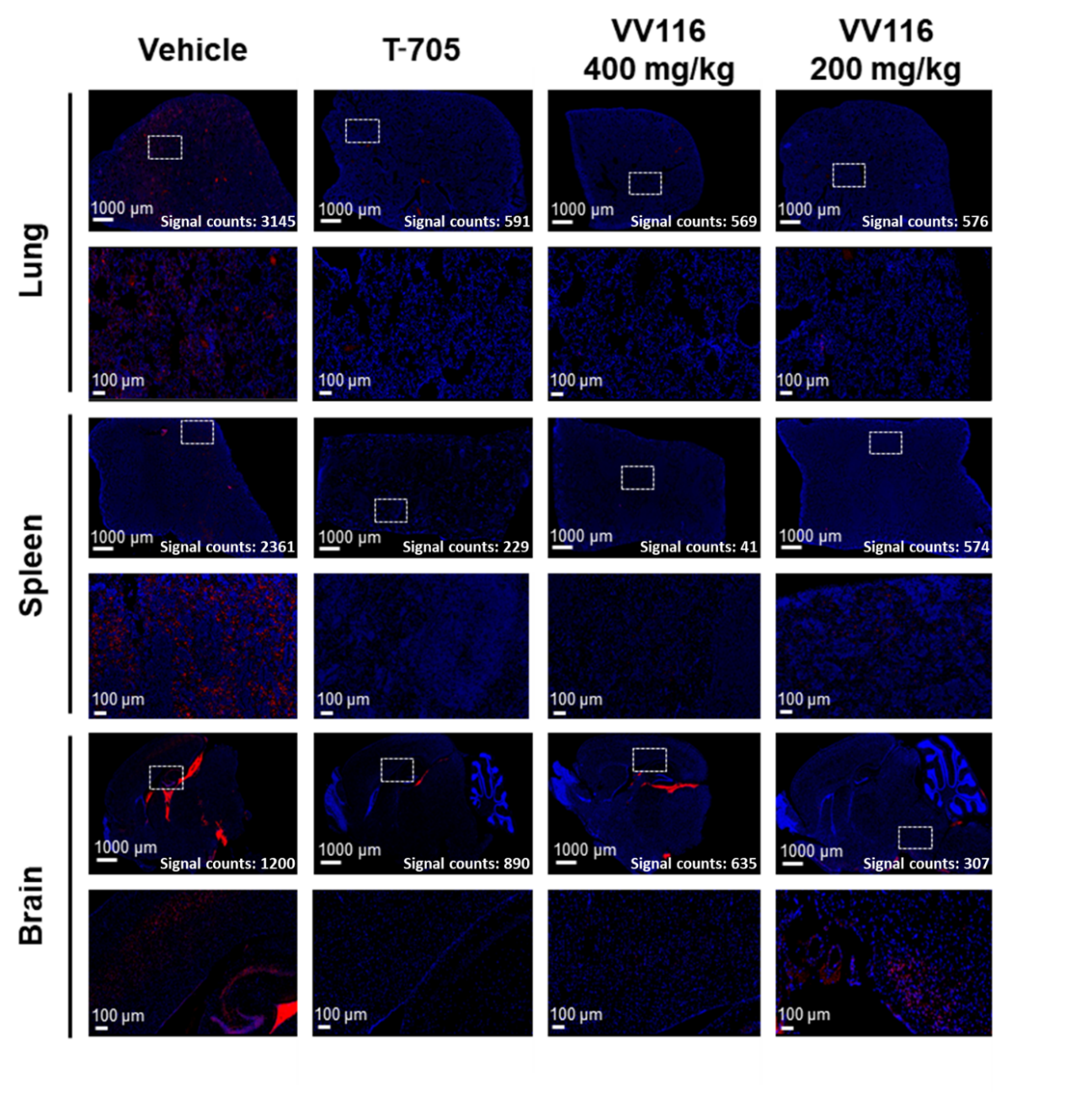
Figure S3** Immunofluorescence staining was performed on hamster tissues to detect the NiV-M antigen (F protein), which appears as discrete red dots. Cell nuclei were stained with DAPI and are shown in blue. Lungs, spleens, and brains of hamsters infected with NiV-M were harvested at 4 d.p.i.. The treatment groups included vehicle control, T-705 (600 mg/kg on the day of viral challenge, followed by 300 mg/kg once daily), and VV116 (400 mg/kg and 200 mg/kg). The discrete red dots representing the viral F protein were quantified as signal counts using ImageJ software (version 1.8, NIH).

**Figure S4** Detection of tissue distribution of X1 in a hamster administrating 400 mg/kg VV116. Data of each time points were collected from three hamsters (*N* = 3) and the error bars denote mean ± standard deviation.

**Table S1** Single-dose PK parameters for X1 in hamsters. Calculation of PK parameters for X1 following oral administration of VV116 at a dose of 400 and 200 mg/kg, respectively (*N* = 3 per group).

| **Group** | **Analyte** | **Hamster #** | **C_max_** | **AUC_0-t_** | **AUC_0-∞_** | **MRT_0-∞_** | **t_1/2_** | **T_max_** |
| --- | --- | --- | --- | --- | --- | --- | --- | --- |
|  |  |  | **(ng/mL)** | **(h*ng/mL)** | **(h*ng/mL)** | **(h)** | **(h)** | **(h)** |
| VV116-400 mg/kg | X1 | 1 | 36400 | 106409 | 112803 | 5.49 | 8.61 | 0.50 |
|  |  | 2 | 51100 | 136784 | 139834 | 3.59 | 6.31 | 0.50 |
|  |  | 3 | 56700 | 129328 | 152676 | 10.97 | 20.23 | 0.50 |
|  |  | Mean | 48067 | 124174 | 135104 | 6.69 | 11.72 | 0.50 |
|  |  | SD | 10484 | 15830 | 20353 | 3.83 | 7.46 | 0.00 |
| VV116-200 mg/kg |  | 4 | 13200 | 45662 | 47512 | 5.33 | 6.22 | 0.50 |
|  |  | 5 | 14600 | 39639 | 41325 | 5.26 | 6.22 | 0.25 |
|  |  | 6 | 15400 | 35013 | 35924 | 4.42 | 5.45 | 0.25 |
|  |  | Mean | 14400 | 40105 | 41587 | 5.01 | 5.96 | 0.33 |
|  |  | SD | 1114 | 5340 | 5798 | 0.50 | 0.45 | 0.14 |
